# Personalized Medicine in the Field of Inflammatory Skin Disorders

**DOI:** 10.3390/jpm12030426

**Published:** 2022-03-09

**Authors:** Mircea Tampa, Monica Neagu, Constantin Caruntu, Simona Roxana Georgescu

**Affiliations:** 1 Department of Dermatology, “Carol Davila” University of Medicine and Pharmacy, 020021 Bucharest, Romania; srg.dermatology@gmail.com; 2 Dermatology Department, “Victor Babes” Hospital of Infectious Diseases, 030303 Bucharest, Romania; 3 “Victor Babes” National Institute of Pathology, 050096 Bucharest, Romania; 4 Colentina University Hospital, 020125 Bucharest, Romania; 5 Department of Physiology, “Carol Davila” University of Medicine and Pharmacy, 050474 Bucharest, Romania

Inflammatory skin diseases occur after the onset of abnormal immune cell responses and the activation of various immune signaling pathways in the skin [1]. Inflammation may become a chronic process when the cause is not removed or the control mechanisms responsible for preventing the progress of the inflammatory process fail. A chronic inflammatory response can lead to cell mutation and proliferation, creating a microenvironment suitable for carcinogenesis [2]. Chronic inflammatory skin diseases are incurable but treatable. Targeted therapies need to be tailored according to the patient’s characteristics. Personalized medicine requires the detection of reliable biomarkers of the pathways that are involved in disease pathogenesis [3]. Recent advances in the field of chronic inflammatory skin diseases have been achieved and are aimed to improve the management of these disorders. A broad range of studies that approach inflammation from several perspectives are available.

Psoriasis is probably the most representative chronic inflammatory skin disease. Psoriasis is a multifaceted and challenging immune disorder with a complex pathogenesis that mainly involves the alteration of the immune system [4]. Nicolescu et al. developed a questionnaire to estimate the national prevalence and risk factors associated with psoriasis. They obtained a 99.13% concordance between the diagnosis based on the questionnaire and the final diagnosis of psoriasis. This is the first study to attempt to identify the prevalence and characteristics of patients with psoriasis in Romania; therefore, these results may represent the starting point for the implementation of new programs including personalized recommendations based on population characteristics [5]. Sobolev et al. found a differential expression of six estrogen-controlled genes (HMOX1, KRT19, LDHA, HSPD1, MAPK1, and CA2) in the skin of female patients with psoriasis compared to male patients with psoriasis and concluded that these proteins may be involved in the defense of skin cells against inflammation damage. Considering that estrogens exert anti-inflammatory effects, low estrogen levels can influence the course of the disease in women with psoriasis [6]. Using a mouse model of induced psoriatic dermatitis, Surcel et al. obtained promising results after the administration of IgY, which led to the improvement in skin lesions and the normalization of immune parameters. IgY is involved in antibacterial and antiviral defense; therefore, these results are important since the skin microbiome participates in the pathogenesis of psoriasis [7].

In the treatment of atopic dermatitis, important advances are being made, and Janus kinase inhibitors (JAK) represent new treatment options. Tsai et al. conducted a systematic review and meta-analysis of the efficacy and safety of JAK inhibitors in atopic dermatitis. The analyzed data showed that JAK inhibitors are an effective therapy, and that side effects are tolerable [8]. This article provides a comprehensive review of the studies available until the time of publication and is welcome given that a few months later, in September 2021, the first topical JAK-1/JAK-2 inhibitor, ruxolitinib, was approved by the FDA, and in early 2022, in January, two oral JAK-1 inhibitors, upadacitinib and abrocitinib, were approved for patients with moderate to severe atopic dermatitis [9].

The identification of novel biomarkers in connective tissue diseases is currently ongoing and may provide new insights into the molecular mechanisms involved in these disorders [10]. Balanescu et al. showed that serum calumenin, S100A6, and cytohesin 2 could be used as biomarkers in systemic sclerosis. Regarding the characteristics of the patients, the most notable association was obtained between the serum levels of these biomarkers and severe skin involvement. Calumenin exhibited the best predictive capacity for cutaneous diffuse manifestation, and S100A6 was correlated with the presence of digital ulcers [11]. Ene et al. emphasized the role of the equilibrium between oxidants and antioxidants in the pathogenesis of lupus nephritis by evaluating markers of oxidative stress according to the four oxidative stress targets—lipids, proteins, nucleic acids, and carbohydrates—and antioxidant capacity. The authors concluded that the oxidant–antioxidant balance is altered in these patients. The most important changes included the impairment of the DNA repair mechanism via 8-oxoguanine DNA glycosylase (OGG1), low serum levels of soluble receptor for advanced glycation end products (sRAGE), which acts as a protective factor against the deleterious effects of inflammation and oxidative stress, the alteration in thiol–disulfide homeostasis, and high nitrotyrosination [12].

The close link between inflammation and skin tumors is well documented [13]. Mediators of inflammation exert pleiotropic effects on the onset and progression of a malignant tumor. Tampa et al. investigated patients with cutaneous squamous cell carcinoma (cSCC) and actinic keratosis and a control group and reported interesting results regarding aberrant sialylation in cSCC, which was associated with tumor aggressiveness. They identified elevated serum levels of total sialic acid (TSA), lipid-bound sialic acid (LSA), ST6GalI, and NEU3 in the cSCC group compared to the control group. Regarding actinic keratoses, only the serum TSA levels were significantly higher. They suggest that the serum ST6GalI/NEU3 level may be useful in differentiating patients with cSCC from noncancer patients [14]. Dudau et al. showed that sea buckthorn seed oil, a compound rich in long-chain fatty acids known to have beneficial effects on the skin, may promote the proliferation of normal and dysplastic keratinocytes under basal conditions and when they are exposed to UVA irradiation. Therefore, this study opens new perspectives on the involvement of lipids in health and disease [15]. Nedelcu et al. focused on recent knowledge regarding the etiology and pathogenesis of halo nevi. Their paper describes clinical aspects, dermoscopic features, immunohistochemical characteristics, and immunological mechanisms involved in the occurrence of halo nevi, which can be viewed as models for better understanding the antitumor human body response [16]. Dobre et al. conducted an extensive review of epigenetic modifications that are involved in the pathogenesis of cutaneous melanoma. In this review, the authors also discuss epigenetic modifications as a source of new therapies for cutaneous melanoma, including cases resistant to conventional therapies, and these data may be used to develop new therapeutic algorithms [17].

The recent literature presents data with clinical significance that can be used to improve the management of various inflammatory skin diseases. There is an increasing body of evidence on the role of cannabinoids in several pathologies [18]. The review by Scheau et al. comprehensively appraises the effects of natural and synthetic cannabinoids on the inflammation of the epithelia of the gut–lung–skin barrier and shows that, although human studies present conflicting data, some results are encouraging and additional studies are required to elucidate the role of cannabinoids in inflammation [19]. Barbu et al. performed an overview of the current status of advanced alginate-based wound dressings for chronic wounds. They present the properties and roles of alginates that make them one of the favorite therapies for chronic wounds. They focused mainly on hydrogels, nanofiber networks, 3D scaffolds and sponges able to capture different cells such as fibroblasts or keratinocytes, or drugs to be released on the wound surface [20]. Georgescu et al. conducted a meta-analysis that investigated the effectiveness of platelet-rich plasma therapy in androgenic alopecia. This is the first meta-analysis that identifies a significant positive correlation between the number of PRP treatments per month and the percentage change in hair density [21]. May et al. reported a challenging case of COVID-19 infection in a patient with pyoderma gangrenosum treated with corticosteroids and high-dose cyclosporine therapy [22].

Our Special Issue underlines personalized medicine as an emerging field of medicine that has the potential to predict which therapy will be safe and efficacious for individual patients using an individual’s genetic profile to guide decisions regarding the diagnosis, treatment, and prevention of disease. Personalized medicine stratifies patients based on molecular markers and allows the identification of the best possible solution for the patient. In this respect, inflammatory skin disorders are complex afflictions with a variable course, treatment response, and unpredictable outcome and represent important candidates for a personalized approach. 
